# Supplementary material for: Risk and rates of hospitalisation in young children: A prospective study of a South African birth cohort
Source: PLOS Glob Public Health. 2024 Jan 17;4(1):e0002754. doi: 10.1371/journal.pgph.0002754 (PMC10793893; doi:10.1371/journal.pgph.0002754)
Supplement: S1 Fig — (PDF) [file pgph.0002754.s001.pdf]

## S1 Fig: DAG model

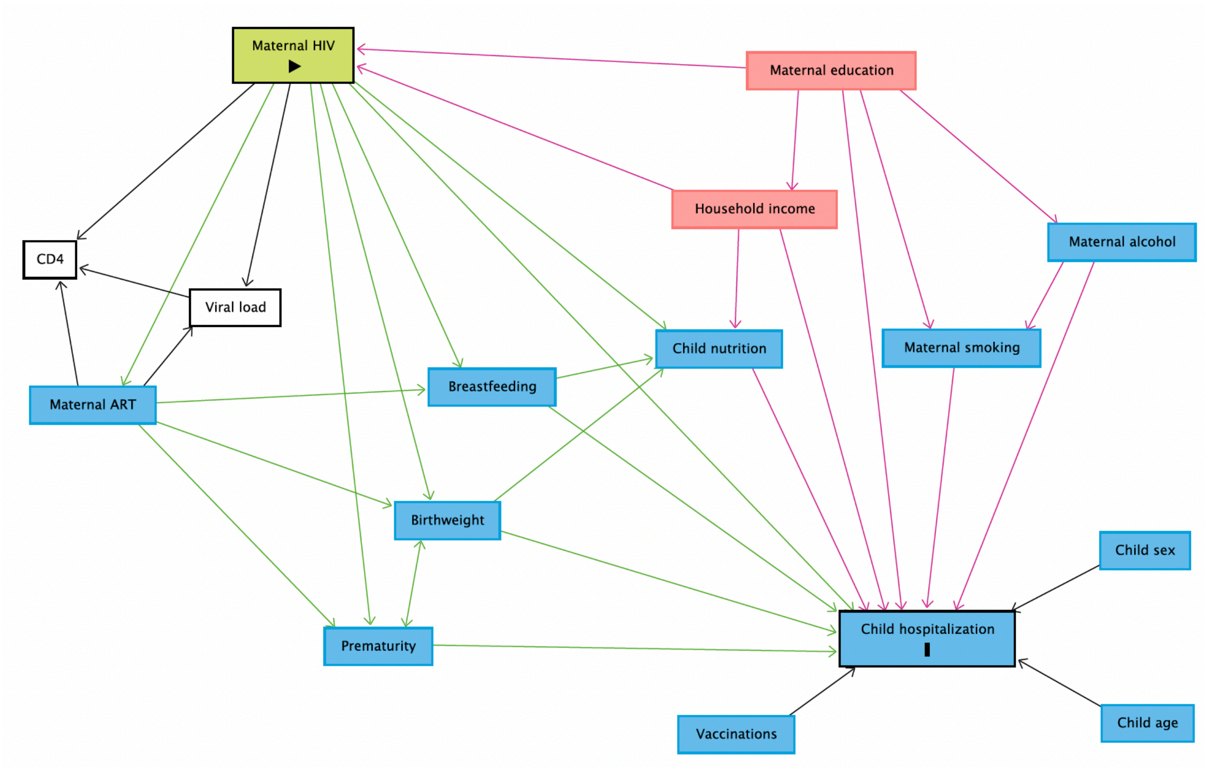


***Footnote:*** Directed Acyclic Graph (DAG) constructed in www.dagitty.net: HIV exposure and child hospitalisation. This DAG was constructed to examine for possible confounding in the relationship between HIV exposure and child hospitalisation from 0 -24 months in the Drakenstein Child Health Study, South Africa. Minimal sufficient adjustment sets for estimating the total effect of maternal HIV exposure on child hospitalisation include household income and maternal education.

***Legend***

- exposure
- outcome
- ancestor of exposure
- ancestor of outcome
- ancestor of exposure *and* outcome
- adjusted variable
- unobserved (latent)
- other variable
- causal path
- biasing path
