# Supplementary material for: Risk and rates of hospitalisation in young children: A prospective study of a South African birth cohort
Source: PLOS Glob Public Health. 2024 Jan 17;4(1):e0002754. doi: 10.1371/journal.pgph.0002754 (PMC10793893; doi:10.1371/journal.pgph.0002754)
Supplement: S2 Fig — (PDF) [file pgph.0002754.s002.pdf]

S2 Fig: Causes of hospitalisation stratified by HIV exposure and age groups

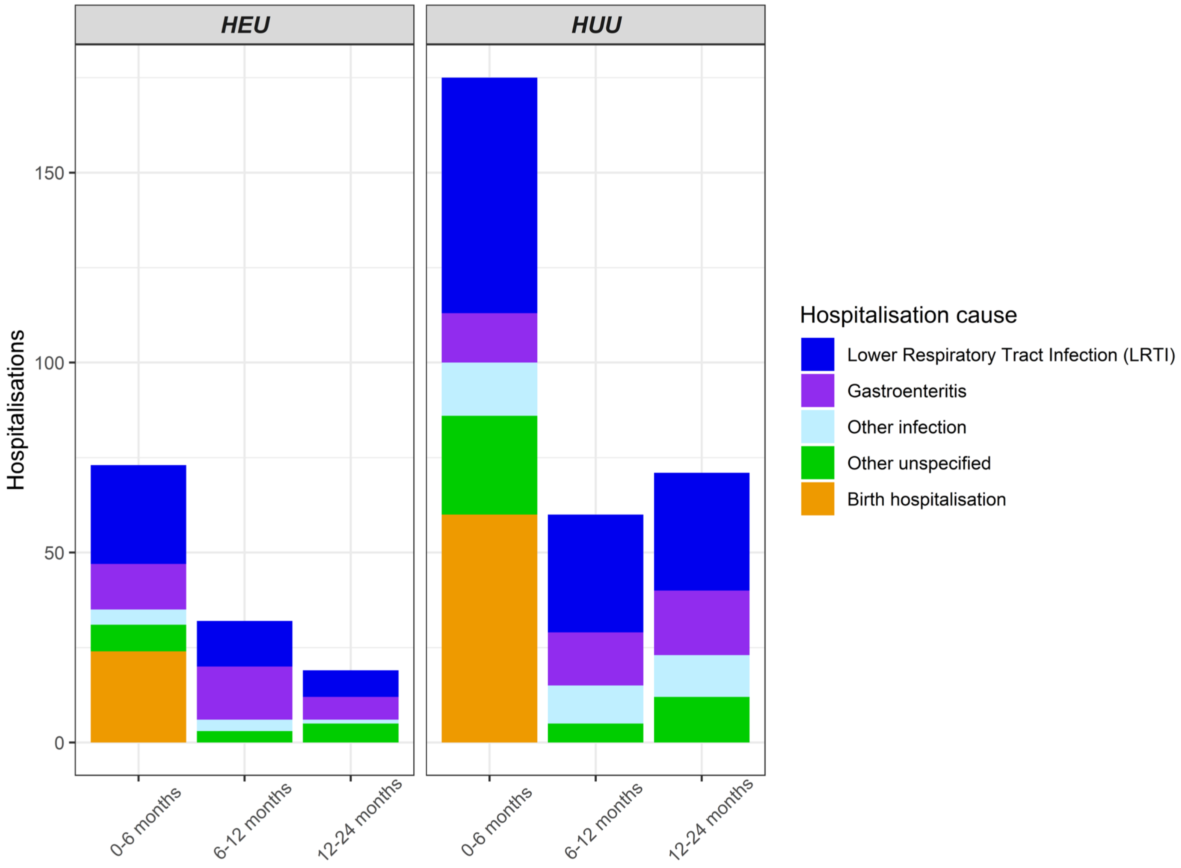

Footnote: Other infections include: meningitis, sepsis, otitis media, urinary tract infection. Other unspecified causes include: Accident and trauma, burns, seizures, nutritional issues (failure to thrive, protein energy malnutrition, micronutrient deficiencies including anaemia)
