# Supplementary material for: Risk and rates of hospitalisation in young children: A prospective study of a South African birth cohort
Source: PLOS Glob Public Health. 2024 Jan 17;4(1):e0002754. doi: 10.1371/journal.pgph.0002754 (PMC10793893; doi:10.1371/journal.pgph.0002754)
Supplement: S1 Table — (PDF) [file pgph.0002754.s003.pdf]

S1 Table: Incidence of hospitalisation in the first two years of life stratified by age

	IR / 1000 person years (95% CI)	IRR (95% CI)
All hospitalisations		
0-12 months		*0-12 v 12-24 months:*
	314 (281-349)	3.81 (2.99-4.85) *
0-6 months		*0-6 v 6-12 months*:
	454 (399-514)	2.69 (2.11-3.44) *
6-12 months		*6-12 v 12-24 months:*
	168 (135-207)	2.04 (1.51-2.76) *
12-24 months		*0-6 v 12-24 months*:
	82 (65-102)	5.51 (4.30-7.70) *

Footnote: IR = Incidence rate; IRR = Incidence rate ratio; HR = Hazard ratio. *p-value < 0.001
