# Supplementary material for: Risk and rates of hospitalisation in young children: A prospective study of a South African birth cohort
Source: PLOS Glob Public Health. 2024 Jan 17;4(1):e0002754. doi: 10.1371/journal.pgph.0002754 (PMC10793893; doi:10.1371/journal.pgph.0002754)
Supplement: S2 Table — (PDF) [file pgph.0002754.s004.pdf]

S2 Table: Proportion of LRTI and RSV-LRTI hospitalisations by age category

	LRTI hospitalizations (n; % of total LRTI hospitalisations [n=169])	RS -LRTI (n; % of total RSV hospitalisations [n=53])	LRTI hospitalisations (n; % of hospitalisations per age group)	RSV-LRTI (n; % of hospitalisations per age group)
**Age category**				
**0-12 months**	131/169 (78%)	46/53 (87%)	131/256 (51%)	46/256 (18%)
**0-6 months**	88/169 (52%)	36/53 (68%)	88/164 (54%)	36/164 (22%)
**6-12 months**	43/169 (25%)	10/53 (19%)	43/92 (47%)	10/92 (11%)
**12-24 months**	38/169 (22%)	7/53 (13%)	38/90 (42%)	7/90 (8%)

Footnote: Numbers exclude birth hospitalisations. Abbreviations: LRTI: Lower respiratory tract infection; RSV: Respiratory syncytial virus
