# Supplementary material for: Risk and rates of hospitalisation in young children: A prospective study of a South African birth cohort
Source: PLOS Glob Public Health. 2024 Jan 17;4(1):e0002754. doi: 10.1371/journal.pgph.0002754 (PMC10793893; doi:10.1371/journal.pgph.0002754)
Supplement: S3 Table — (PDF) [file pgph.0002754.s005.pdf]

S3 Table: Incidence of hospitalisations in the first two years of life by HIV exposure status excluding recurrent events

	HEU IR / 1000 person years	HUU IR /1000 person years	IRR (95% CI)
All hospitalisations			
0-12 months	334 (262-418)	223 (192-258)	1.49 (1.14-1.95) **
0-6 months	510 (388-658)	349 (295-410)	1.46 (1.08-1.97) *
6-12 months	144 (82-234)	89 (62-123)	1.62 (0.90-2.92)
12-24 months	50 (25-90)	45 (31-62)	1.12 (0.57-2.20)

Footnote: ** p-value < 0.01; * p-value < 0.05. Abbreviations: HEU = HIV-exposed uninfected; HUU = HIV-unexposed uninfected; IR = Incidence rate; IRR = Incidence rate ratio
