# Supplementary material for: Risk and rates of hospitalisation in young children: A prospective study of a South African birth cohort
Source: PLOS Glob Public Health. 2024 Jan 17;4(1):e0002754. doi: 10.1371/journal.pgph.0002754 (PMC10793893; doi:10.1371/journal.pgph.0002754)
Supplement: S4 Table — (PDF) [file pgph.0002754.s006.pdf]

S4 Table: Incidence of hospitalisations in the first two years of life by HIV exposure status excluding birth hospitalisations

				Unadjusted	***Adjusted model 1***	***Adjusted model 2***
	**All IR / 1000 person years**	**HEU IR / 1000 person years**	**HUU IR /1000 person years**	**IRR (95% CI)**	**HR (95% CI)**	**HR (95% CI)**
0-12 months	235 (207-266)	346 (274-431)	205 (175-238)	1.69 (1.30-2.21) ***	1.53 (1.14-2.07) **	1.59 (1.16-2.18) **
0-6 months	299 (255-349)	424 (314-559)	265 (219-319)	1.60 (1.14-2.23) **	1.48 (1.03-2.12) *	1.56 (1.07-2.28) *
6-12 months	168 (135-207)	265 (179-379)	142 (107-183)	1.87 (1.20-2.91) **	1.73 (1.05-2.85) *	1.76 (1.00-3.08) *
12-24 months	82 (65-102)	77 (45-124)	84 (65-106)	0.92 (0.54-1.57)	0.91 (0.53-1.55)	0.78 (0.44-1.38)

Footnote: Unadjusted incident rate ratios and adjusted hazard models for hospitalisations in the first 2 years of life by HIV exposure excluding birth hospitalisations.
IR = Incidence rate; IRR = Incidence rate ratio; HR = Hazard ratio. Multivariate models adjusted for (1) maternal education and household income; (2) maternal education, household income, maternal age at birth and maternal smoking. *** p-value < 0.001; ** p-value < 0.01; * p-value < 0.05
Abbreviations: HR = Hazard ratio; HEU = HIV-exposed uninfected; HUU = HIV-unexposed uninfected; IR = Incidence rate; IRR = Incidence rate ratio; OR = Odds ratio
