# Supplementary material for: Risk and rates of hospitalisation in young children: A prospective study of a South African birth cohort
Source: PLOS Glob Public Health. 2024 Jan 17;4(1):e0002754. doi: 10.1371/journal.pgph.0002754 (PMC10793893; doi:10.1371/journal.pgph.0002754)
Supplement: S5 Table — (PDF) [file pgph.0002754.s007.pdf]

S5 Table: Impact of birth, breastfeeding and HIV-related factors on hospitalisations stratified by HIV exposure status and age excluding birth hospitalisations

	HEU<br>HR (95% CI)	HUU<br>HR (95% CI)	Total<br>HR (95% CI)
Birth factors			
Prematurity			
0-12 months	1.94 (1.11-3.37) *	1.75 (1.22-2.52) **	1.85 (1.36-2.52) ***
0-6 months	1.67 (0.89-3.13)	2.04 (1.34-3.11) ***	1.95 (1.37-2.78) ***
6-12 months	2.66 (1.07-6.62) *	1.26 (0.66-2.41)	1.74 (0.99-3.04)
12-24 months	1.48 (0.43-5.11)	0.97 (0.45-2.06)	1.07 (0.56-2.04)
Feeding			
Ever breastfed			
0-12 months	0.85 (0.51-1.43)	0.76 (0.42-1.39)	0.63 (0.46-0.87) **
12-24 months	1.00 (0.39-2.62)	1.07 (0.35-3.22)	1.08 (0.60-1.94)
Breastfeeding for a year			
0-12 months	1.02 (0.39-2.62)	0.71 (0.50-1.02)	0.67 (0.48 -0.93) *
12-24 months	0.97 (0.24-3.88)	0.64 (0.36-1.11)	0.70 (0.42 -1.16)
Immunization timing by 9 months			
0-12 months	1.48 (0.87-2.52)	1.36 (0.96-1.92)	1.39 (1.04-1.87) *
12-24 months	1.34 (0.52-3.48)	1.03 (0.60-1.77)	1.09 (0.68-1.75)
HIV-related variables			
CD4 - categorical *(>500 vs <=500)*			
0-12 months	0.99 (0.56-1.76)	-	-
0-6 months	0.92 (0.46-1.87)	-	-
6-12 months	1.11 (0.43-2.83)	-	-
12-24 months	0.88 (0.33-2.35)	-	-
Viral load-categorical *(>= 40 vs <40)*			
0-12 months	1.51 (0.79-2.86)	-	-
0-6 months	0.92 (0.43-1.98)	-	-
6-12 months	4.43 (1.52-12.87) **	-	-
12-24 months	1.31 (0.40-4.30)	-	-
ART regimen initiation *(Before vs during pregnancy)*			
0-12 months	0.86 (0.51-1.44)	-	-
0-6 months	1.06 (0.58-1.93)	-	-
6-12 months	0.54 (0.20-1.40)	-	-
12-24 months	0.76 (0.29-1.99)	-	-

Footnote: Results with birth hospitalisations excluded. *** p-value < 0.001; ** p-value < 0.01; * p-value < 0.05 for unadjusted models. Definitions: Prematurity (<37 vs >= 37 weeks); Ever breastfeeding (>0 months vs = 0 months); Breastfeeding for a year (>11 months vs <= 11 months). Abbreviations: ART = antiretroviral therapy; HR = Hazard ratio; HEU = HIV-exposed uninfected; HUU = HIV-unexposed uninfected
