# Supplementary material for: Risk and rates of hospitalisation in young children: A prospective study of a South African birth cohort
Source: PLOS Glob Public Health. 2024 Jan 17;4(1):e0002754. doi: 10.1371/journal.pgph.0002754 (PMC10793893; doi:10.1371/journal.pgph.0002754)
Supplement: S6 Table — (PDF) [file pgph.0002754.s008.pdf]

S6 Table: Association between malnutrition and hospitalisation

	Underweight			
	**Total**	**HEU**	**HUU**	**OR (95% CI)**
0-12 months	47 (19%)	19 (24%)	28 (17%)	1.62 (0.83-3.12)
0-6 months	35 (22%)	14 (29%)	21 (19%)	1.76 (0.80-3.85)
6-12 months	12 (14%)	5 (17%)	7 (12%)	1.46 (0.40-5.03)
12-24 months	9 (11%)	4 (25%)	5 (7.6%)	4.07 (0.90-17.69)
	**Stunted**			
0-12 months	71 (31%)	27 (38%)	44 (28%)	1.63 (0.90-2.94)
0-6 months	47 (32%)	16 (38%)	31 (30%)	1.45 (0.68-3.06)
6-12 months	24 (28%)	11 (38%)	13 (23%)	2.07 (0.78-5.51)
12-24 months	14 (18%)	4 (27%)	10 (16%)	2.41 (0.65-8.32)
	**Wasted**			
0-12 months	29 (15%)	11 (19%)	18 (14%)	1.50 (0.64-3.37)
0-6 months	18 (17%)	8 (28%)	10 (13%)	2.51 (0.86-7.22)
6-12 months	11 (13%)	3 (10%)	8 (14%)	0.71 (0.15-2.68)
12-24 months	8 (10%)	3 (20%)	5 (7.9%)	2.90 (0.54-13.55)

Abbreviations: HEU = HIV-exposed uninfected; HUU = HIV-unexposed uninfected; OR = Odds ratio
