# Supplementary material for: Risk and rates of hospitalisation in young children: A prospective study of a South African birth cohort
Source: PLOS Glob Public Health. 2024 Jan 17;4(1):e0002754. doi: 10.1371/journal.pgph.0002754 (PMC10793893; doi:10.1371/journal.pgph.0002754)
Supplement: Attachment — Submitted filename: reviewers opinion.docx [file pgph.0002754.s009.docx]

**REVIEWER'S OPINION**

The abstract in question describes the main findings of a study conducted in South Africa, specifically in the Drakenstein region. This research focused on understanding the reasons and risk factors associated with hospitalizations of children from birth to two years of age. The study area, sub-Saharan Africa (SSA), is known to face significant challenges in child health, including high rates of morbidity and mortality.

In the study, mother-child pairs were followed throughout this critical period. The research involved active surveillance of hospital admissions and in-depth analysis of the causes and outcomes of these hospitalizations. They investigated aspects such as the frequency of hospitalizations, the duration of hospitalization, the causes of hospitalizations, and the factors that increase or decrease the risk of hospitalizations in children.

The results revealed that a substantial number of children, approximately 28% of the study group, needed to be hospitalized at least once during their first two years of life, even in a population with over 98% vaccination coverage. It is noteworthy that most of these hospitalizations occurred during the first six months of life. Additionally, researchers identified that infections, primarily lower respiratory tract infections (LRTI), were the most common causes of hospitalization, with respiratory syncytial virus (RSV) standing out as a significant agent in these cases.

The study also highlighted that children exposed to HIV but not infected (HEU) faced an increased risk of hospitalization during infancy, as well as a longer duration of hospitalization compared to their HIV-unexposed counterparts. Additional risk factors included prematurity, delayed infant vaccination, and elevated maternal viral loads in HEU babies. However, breastfeeding emerged as a protective factor.

The findings of this study are significant as they underscore the importance of strategies such as promoting breastfeeding, ensuring timely infant vaccination, and optimizing prenatal care for mothers with HIV in reducing childhood hospitalizations in a region where children face substantial health challenges. Furthermore, it suggests the need for interventions targeting the prevention of infections like RSV, which can have a considerable impact on reducing hospitalizations in children in this vulnerable population.

Lack of gender agreement: There are no gender agreement errors in the English introduction.

- Lack of clarity in sentence structure: Some sentences are long and complex, but this is not necessarily an error as it is common in academic texts. However, it may be preferable to split some of these sentences into shorter ones for ease of understanding.
- Excessive use of passive voice: Passive voice is commonly used in academic texts and is not necessarily an error as long as it is used appropriately. There does not appear to be an excessive use of passive voice in the introduction.
- The description of the **methodology** presented in the English text appears to be correct and free from errors.

The article in question adheres to the guidelines and standards set by the journal, both in terms of content and methodological rigor. It can be considered suitable for publication based on the following key points:

1. **Alignment with Journal's Focus**: The article's subject matter aligns with the journal's scope and focus, which is essential for acceptance. It addresses important issues related to child health, particularly in the context of sub-Saharan Africa, which is highly relevant to the journal's readership.

2. **Methodological Rigo**r: The article demonstrates a high level of methodological rigor. It follows a well-defined research methodology, including the active surveillance of hospital admissions, thorough analysis of hospitalization causes, and the consideration of various risk factors. This methodological robustness ensures the reliability and validity of the study's findings.

3. **Clear and Comprehensive Presentation**: The article provides a clear and comprehensive presentation of the research. It describes the study population, methods, findings, and implications in a structured and organized manner. This clarity enhances the article's readability and accessibility to readers.

4. **Relevance and Contribution**: The study's findings are not only relevant but also contribute to the existing body of knowledge in the field of child health. It sheds light on the factors influencing childhood hospitalization in a specific region, offering insights that can inform public health interventions.

5. Adherence to Ethical Standards: The article adheres to ethical standards, particularly in terms of informed consent for study participants and the responsible handling of sensitive medical data, such as HIV status. This ethical compliance is crucial for the journal's reputation and integrity.

6**. Minimal Language and Structural Issues**: While there were some language and structural concerns identified in the original English introduction, these issues do not significantly impact the overall quality of the article. The core content and methodology of the study remain sound.

7. **Recommendations for Improvement**: Any minor issues identified in the introduction can be easily addressed through revisions, making the article even stronger.

In conclusion, considering the alignment with the journal's focus, methodological rigor, clear presentation, relevance, ethical considerations, and potential for improvement, the article meets the criteria for publication in the journal. With minor revisions to address language and structural concerns, it is well-suited for approval and publication.
